# Formulation and preclinical studies with a trivalent rotavirus P2-VP8 subunit vaccine

**DOI:** 10.1080/21645515.2019.1710412

**Published:** 2020-01-29

**Authors:** Kyle Lakatos, David McAdams, Jessica A. White, Dexiang Chen

**Affiliations:** Medical Devices and Health Technologies Global Program, Formulation Technologies, PATH, Seattle, WA, USA

**Keywords:** Rotavirus, nonreplicating rotavirus vaccine (NRRV), antigen adsorption, antigen integrity, immunogenicity

## Abstract

More effective rotavirus vaccines are essential for preventing extensive diarrheal morbidity and mortality in children under five years of age in low-resource regions. Nonreplicating rotavirus vaccines (NRRV) administered parenterally provide an alternate vaccination method to the current licensed oral vaccine. Live attenuated vaccines and may generate increased efficacy in low-resource settings because the parenteral administration route bypasses some of the challenges associated with oral administration, including differences in intestinal environments. Work described here supports development of a trivalent NRRV vaccine for parenteral administration to avoid complications of the gastrointestinal route. Recombinant VP8* subunit proteins representing some of the most prevalent strains of rotavirus infecting humans – DS-1 (P[4]), 1076 (P[6]), and Wa (P[8]) – were combined with an aluminum adjuvant and the P2 epitope of tetanus toxoid to enhance the immune response to this NRRV antigen. Vaccine formulation development included selection of aluminum hydroxide (Alhydrogel®) as an appropriate adjuvant as well as an optimal buffer to maintain antigen stability and optimize antigen binding to the adjuvant. Characterization assays were used to select the lead vaccine formulation and monitor formulation stability. The NRRV liquid formulation was stable for one year at 2°C to 8°C and four weeks at 37°C. Immunogenicity of the NRRV formulation was evaluated using a guinea pig model, where we demonstrated that the adjuvant provided a 20-fold increase in neutralization titer against a homologous antigen and that the P2-fusion also enhanced the serum neutralizing antibody responses. This vaccine candidate is currently being evaluated in human clinical trials.

## Introduction

Rotavirus is a leading cause of severe diarrheal disease worldwide in children under five years of age, with infections resulting in 146,000 to 215,000 deaths annually, mostly in low- and lower-middle-income countries.^1,2^ To address the significant morbidity and mortality caused by rotavirus, multiple vaccines have been or are being developed and licensed for use.^3^ All of the licensed rotavirus vaccines are oral, live attenuated formulations, and two – Rotarix® (GlaxoSmithKline) and RotaTeq® (Merck) – have support from Gavi, the Vaccine Alliance for use in low-income countries. Recently, two additional oral vaccines – Rotavac® (Bharat Biotech) and Rotasil® (Serum Institute of India) – were prequalified by the World Health Organization.^4,5^ Additional rotavirus vaccines have been developed but currently are limited to use in the countries of production and not available on the international market: Rotavin-M1 in Vietnam and Lanzhou lamb in China.^6,7^

Despite introduction of several vaccines, rotavirus is still a major cause of gastroenteritis and diarrhea worldwide.^8^ Several factors have been identified as contributing to the rotavirus disease burden in low-income countries, including reduced vaccine efficacy (~50%) compared with that in middle- and high-income countries. (>90%).^6,9-11^ The cause of this dramatic difference in efficacy is unknown; however, several potential causes have been identified, including higher rotavirus transmission rates; differences in intestinal environments (e.g., microbiome, coinfection); and host mucosal factors (e.g., presence of breast milk constituents at the time of immunization, histo-blood group antigens, and Lewis secretor antigens).^9,10,12^ Alternative approaches such as nonreplicating rotavirus vaccine (NRRV) candidates administered via the parenteral route may provide higher efficacy compared with oral rotavirus vaccination in these settings by eliminating the complications of the gastrointestinal route.^13,14^ Parenteral vaccines may be manufactured at lower cost and could be delivered along with routine Expanded Program on Immunization vaccines via established networks, further reducing costs.^15^

Rotavirus virions are double-stranded RNA particles classified by six nonstructural proteins (NSP1–NSP6) and six structural proteins (VP1–VP4, VP6 and VP7) which form a three-layered structure of inner core, inner capsid, and outer capsid.^16,17^ During infection, VP4 is cleaved via proteolysis into VP8* and VP5* fragments, which are required for cellular infection.^18^ The rotavirus strains that most frequently infect humans can be grouped into three P genotypes, P[4], P[6], and P[8], each defined by slightly different VP8* protein structures.^19,20^

Several rotavirus vaccines are being developed for parenteral administration as alternatives to existing oral rotavirus vaccines. These include a heat-inactivated vaccine; VP6-expressed protein vaccines from the inner capsid protein; virus-like particle vaccines; and the candidate discussed in this manuscript, a trivalent recombinant VP8* subunit vaccine.^15^ Formulation development for this trivalent recombinant subunit VP8* vaccine, an NRRV for parenteral administration, is presented here. Recombinant VP8* proteins for human rotavirus strains DS-1 (P[4]), 1076 (P[6]), and Wa (P[8]) were selected to provide protection against the most prevalent strains of rotavirus infecting humans.^20^ These recombinant protein antigen candidates were fused with the P2 epitope of tetanus toxoid,^21^ and adsorbed onto an aluminum salt suspension based adjuvant (Alhydrogel® or AdjuPhos®) to improve immunogenicity consistent with several licsenced vaccines.^22^ The P2 epitope induces a strong T cell helper function and has been shown to enhance the immunogenicity of the VP8* recombinant antigens in mouse and guinea pig models.^21^ This is the most advanced NRRV candidate and data presented here describe formulation development to support ongoing study with this vaccine candidate. Here we report formulation, stability, and preclinical immunogenicity studies for this trivalent P2-VP8* candidate NRRV.

## Materials and methods

### Rotavirus antigen

Bulk monovalent constructs P2-VP8* P[4] (DS-1 strain), P2-VP8* P[6] (1076 strain), and P2-VP8* P[8] (Wa strain) produced in *Escherichia coli* (*E. coli*) were prepared and purified at Walter Reed Army Institute of Research as published previously.^13,23^ Briefly, a synthetic gene, codon optimized for *E. coli*, was inserted into the pJ411 expression plasmid under control of a T7 promoter (optEc2) (DNA 2.0). The plasmid was transfected into *E. coli* Bl21 (DE3) (Novagen). Master and working seed lots as well as final vaccine were produced. Protein was purified by physically disruption of cells followed by centrifugation and then filtration using a Q Sepharose FF column (GE Healthcare) collecting the protein in the flow through.

### Adjuvant adsorption capacity

Adsorption capacity of two aluminum-based adjuvants, Adju-Phos and Alhydrogel (InvivoGen; Accurate Chemical & Scientific Corporation, #APO2222 and #A1090) were tested using monovalent P2-VP8* P[8] antigen. A two-fold working stock suspension (2.25 mg Al/mL) of each adjuvant was prepared in 0.9% (w/v) saline (Teknova, #S5815). A 240 µg/mL P2-VP8* P[8] working stock was prepared in saline. The P2-VP8* P[8] working stock was mixed with each aluminum adjuvant and diluted with saline to achieve final antigen concentrations of 120, 60, or 20 µg/mL in suspension with adjuvant particles (Alhydrogel or Adju-Phos) at 1.125 mg Al/mL. Formulations were prepared in triplicate and the percent adsorption was evaluated using the Pierce™ bicinchoninic acid (BCA) assay (Thermo Fisher Scientific™, #23225) for protein content. Samples were taken for analysis at t = 0, t = 6 hours at room temperature (RT), and t = 24 hours (30 minutes at RT followed by overnight inversion at 2°C to 8°C). A 0.2 mL portion of the sample was drawn for analysis of total sample protein. Free or unbound protein samples were prepared by centrifuging at 1,500 x g for five minutes to pellet the aluminum adjuvant and collecting the supernatant containing the unbound protein. Pellets – containing the aluminum adjuvant and any bound antigen – were resuspended in saline for analysis. After samples were mixed with BCA working reagent, they were assayed in 96-well plates according to kit instructions. Sample protein content was analyzed in comparison with bovine serum albumin (BSA; Roche Diagnostics, #3117332001) protein standards, and the degree of adsorption was measured by the percent bound protein according to the following equation:

$$\%\;bound\;protein=\frac{Protein\;in\;pellet\;fraction\;\left(\frac{mg}{mL}\right)}{Total\;protein\;\left(\frac{mg}{mL}\right)}*100\%$$

The effect of the phosphate (PO_4_) buffer concentration on antigen adsorption to adjuvant also was determined by using the P2-VP8* P[8] antigen alone. The percent adsorption of P2-VP8* P[8] (120 µg/mL) to Alhydrogel (1.125 mg Al/mL) was measured in buffer concentrations of 0, 0.4, 1.7, and 6.7 mM PO_4_ in saline, prepared by dilution of stock (11.9 mM PO_4_) phosphate buffered saline (Growcells.com, #MRGF-6230) pH 7.2 with 0.9% (w/v) saline (Teknova, #S5815). Samples were measured at 0 and 24 hours after adsorption at 2°C to 8°C. The experiment was repeated three times for each buffer concentration.

The percent adsorption for each monovalent antigen as well as for trivalent P2-VP8* antigens to Alhydrogel was evaluated in the presence of 1.0 mM and 0.5 mM PO_4_ buffer for concentrations ranging from 30 to 360 µg/mL of protein, using the BCA assay as described above.

### Trivalent P2-VP8 formulation development and characterization

A phosphate buffered saline solution was prepared by diluting stock buffer with saline to produce a low PO_4_ buffer, 0.5 mM PO_4_. A two-fold working stock of the trivalent P2-VP8* formulation was prepared by mixing equal volumes of each of the monovalent antigens at 240 µg/mL in 0.5 mM PO_4_ in saline for a final solution containing 720 µg/mL P2-VP8* trivalent antigens. Equal volumes of this antigen mixture and a suspension of Alhydrogel (2.25 mg Al/mL diluted in 0.5 mM PO_4_ in saline) were combined using gentle mixing overnight at 2°C to 8°C to achieve a formulation with 360 µg/mL total trivalent P2-VP8* antigen containing Alhydrogel at 1.125 mg Al/mL in 0.5 mM PO_4_ (pH 7.2). The final adjuvanted trivalent vaccine formulation was filled into sterile 2 mL glass serum vials (Wheaton®, #223683), crimp sealed, and stored at 2°C to 8°C for long-term stability testing. Additional vials of the formulation were stored at 25°C and 37°C for accelerated stability testing for three months.

Formulation appearance was assessed by visual inspection of the even distribution of particulates and the color of the suspension. The pH was measured with a pH microprobe (InLab® UltraMicro pH probe, Mettler Toledo, #51343163). Measurements were taken in triplicate and the average result was reported.

Ion exchange high-performance liquid chromatography (IEX-HPLC) was used to confirm antigen identities in the trivalent formulation at t = 0. Antigens were desorbed from the adjuvant in the formulation by increasing the PO_4_ buffer concentration to 20 mM (pH 7.5), followed by a one-hour incubation at RT. Samples were centrifuged at 1,500 x g for five minutes, and the desorbed antigen in the supernatant was transferred to HPLC vials for analysis. Monovalent standards and a trivalent control were prepared from bulk material at the same theoretical concentration as the test sample. The HPLC system was set up with a J.T.Baker® cation exchange column (Bakerbond® wide-pore Carboxy-Sulfon, VWR® International, #7159-00); mobile phase A was 20 mM sodium phosphate adjusted to pH 5.65 ± 0.1, and mobile phase B was 20 mM sodium phosphate and 250 mM sodium chloride, adjusted to pH 5.65 ± 0.1. The column temperature was 40°C and the in-line ultraviolet detector was set to record the absorption at 280 nm. The flow rate was set at 1 mLl/min for the first 30 minutes of the method, then transitioned to 2 mL/min for minutes 30 to 36 and then transitioned back to 1 mL/min for the remainder of the method. A mobile phase gradient of 5% B (0–2 min), 5→100% B (2–30 min), 100% B (30–36 min), 100→5% B (37 min), and 5% B (37–50 min) was used.

Samples were tested in triplicate along with monovalent standards and the trivalent control. The average elution times of the major peak for each of the monovalent standards were recorded and compared with the average elution times for the corresponding major peaks from the trivalent sample chromatograms. The sample chromatogram was compared with the trivalent control chromatogram to ensure that no anomalous peaks were present.

Antigen integrity was determined by sodium dodecyl sulfate polyacrylamide gel electrophoresis (SDS-PAGE) after desorption from the aluminum adjuvant. Test samples were desorbed from the aluminum adjuvant by mixing samples to achieve a final concentration of 20 mM PO_4_. Sampels were then vortexed and incubated for 1 hour at room temperature. After incubation test samples were divided into whole vaccine, supernantant, and pellet aliquots for measurement using centrifugation at 1,500 x g for five minutes. Test samples from the one-year stability study required modifications to the above described desorption method. These modifications were to incubate with a mixture of 2.5% w/v SDS and 20 mM PO_4_ buffer and heat to 95°C for ten minutes in place of the PO_4_ buffer alone at room temperature.^24^

After desorption samples were prepared at 200 µg/mL with NuPAGE™ lithium dodecyl sulfate sample buffer (Thermo Fisher Scientific, #NP0007) and reducing agent and heated at 70°C for ten minutes, per the manufacturer’s protocol. Prepared samples (10 µL) were loaded into the wells of a 4% to 20% gradient gel in NuPAGE 2-(N-morpholino)ethanesulfonic acid running buffer (Thermo Fisher Scientific, #NP0002), and gels were run at 200 mV for 35 minutes, followed by staining for one hour with GelCode™ blue stain (Thermo Fisher Scientific, #24590). Gels were destained for five minutes in ultrapure water and images were captured using an AlphaImager® (previously Alphametrics Alpha Innotech, now ProteinSimple). The passing criterion for the formulation was presence of two bands near 20 kDa, with higher intensity in the lower band.

Antigen adsorption and total protein content were measured using absorption at 280 nm (A280) and BCA. For A280 testing, samples were vortexed and centrifuged at 1,500 x g for five minutes. The absorbance of a 0.2 mL sample of supernatant was measured on a Shimadzu UV-1800 UV-VIS spectrophotometer (Shimadzu Scientific Instruments; BRAND® UV cuvettes, Sigma-Aldrich, #Z628026). BCA was used to measure total protein in the whole vaccine, the supernatant, and the pellet (portion bound to adjuvant). For measurements of protein in the whole vaccine, a sample was vortexed and 75 µL was withdrawn and mixed with 600 µL of BCA working reagent. For measurements of protein in the supernatant and the pellet, 500 µL samples were centrifuged at 16,000 x g for five minutes. Next, 420 µL of supernatant was removed and from this, 75 µL was used in the measurement of protein in the supernatant by mixing with 600 µL of BCA working reagent. The pellet was reconstituted with 420 µL of 0.5 mM PO_4_ in saline, and a 75 µL aliquot was mixed with 600 µL of BCA working reagent. Test samples were assayed in triplicate in 96-well plates according to kit instructions.

Aluminum content was quantified by a chelation assay.^25^ Briefly, a sample of the formulation was mixed with concentrated sulfuric acid (Sigma-Aldrich #209198), heated, and neutralized with sodium hydroxide (Macron Fine chemicals #7680-04). Acetate buffer (8.20 ± 0.07 g sodium acetate (Sigma-Aldrich #32318) and 7.71 ± 0.06 g ammonium acetate (Sigma #A-1542) into 60 mL of 18 MΩ dH_2_O) and EDTA (Avantor #8991-01) were added, and the mixture was gently boiled, with stirring, for five minutes to ensure complete complexing of the aluminum with EDTA. The excess EDTA then was bound by adding cupric sulfate (Sigma-Aldrich #209198) in the presence of pyridylazonaphthol indicator (Sigma-Aldrich #101036), and the aluminum content of the sample determined by titration.

### Stability studies

After selection of adjuvant and vaccine buffer long-term stability studies (2°C to 8°C) were conducted using the methods described above. Formulated trivalent vaccine was aliquoted into 0.7 mL in glass vials and placed at 2°C to 8°C. Samples were collected and tested over 1 year.Target product attributes defined for the final NRRV vaccine formulation were used to monitor formulation stability.

### Animal studies

Two animal immunogencity studies were conducted at Noble Life Sciences, Inc. Both animal studies complied with the Animal Welfare Act regulations (9 US Code of Federal Regulations); the US Public Health Service Policy on Humane Care and Use of Laboratory Animals;^26^ the Guide for the Care and Use of Laboratory Animals;^27^ and the Association for Assessment and Accreditation of Laboratory Animal Care International accreditation.

In the first study, the effect of the aluminum adjuvant on monovalent P2-VP8* P[8] immunogenicity was evaluated using 15 three-week-old male guinea pigs weighing at least 300 g (Hartley strain; Elm Hill Laboratories). P2-VP8* P[8] with Alhydrogel was administered to six animals as a 0.25 mL injection containing 30 µg antigen and 430 µg adjuvant into the quadriceps muscle on days 0, 14, and 28. Another six animals received 30 µg of P2-VP8* P[8] antigen without adjuvant, and three control animals were not injected. Animals were assessed for pain at 24, 48, and 72 hours post dosing. Blood was collected on days 0 and 42 (two weeks after the third immunization). The day 42 blood samples were tested for binding immunoglobulin G (IgG) antibody responses and neutralizing antibody responses against both the homologous vaccine antigen, Wa P[8], used for immunization, as well as the heterologous vaccine antigen strains.^28^

A second study was conducted to evaluate the effect of P2-fusion using male and female guinea pigs weighing from 300 to 350 g (Hartley strain; Charles River Laboratory). Animals were divided into seven groups, each containing five female and four male animals. P2-fused and non-fused VP8* antigens adjuvanted with Alhydrogel were administered as a 0.25 mL injection containing 30 µg antigen and 280 µg adjuvant into the quadriceps muscle on days 0, 14, and 28. Animals were assessed for pain at 24, 48, and 72 hours post dosing. Blood was collected on days 0, 14, 28, and 42. Blood samples were tested for total binding IgG antibody responses and neutralizing antibody responses on day 42 as described above. Total binding IgG responses were evaluated using both P2-fused and non-fused antigens to coat enzyme-linked immunosorbent assay (ELISA) plates. One animal died from complications with anesthesia or blood draw on day 1.

Neutralizing antibodies were evaluated prior to immunization on day 1 and day 42 according to previously published methods (Cincinnati Children’s Hospital Medical Center).^29^ Neutralizing antibodies were screened against strains DS-1 (P[4]) and Wa (P[8]). In the first study, an alternate strain of P[6], ST3, was used to screen for neutralizing antibodies, rather than 1076.

Total binding IgG anti-P2-VP8* P[8] antibodies also were evaluated at day 42. For this assessment, Corning® high-binding 96-well ELISA plates (Fisher Scientific, #07-200-39) were coated with antigen diluted in HyClone™ Dulbecco’s posphate buffered saline (DPBS; Fisher Scientific, #SH3002803). Plates were sealed and incubated overnight for 18 to 24 hours at 2°C to 8°C. Plates were washed by adding 300 µL of wash buffer (DPBS with 0.05% Tween™ 20, Fisher Scientific, #BP337-100) per well, aspirating and repeating for a total of three wash cycles before removing all remaining wash buffer. Blocking buffer (DPBS with 1% BSA) was added at 200 µL per well and plates were sealed and incubated for one to four hours at RT. Serial dilutions of test sera samples and positive and negative controls were prepared in assay buffer (DPBS with 1% BSA and 0.05% Tween 20) in deep well plates (VWR, #82006-448). ELISA plates were washed as described above and 100 µL of each prepared dilution was transferred from the deep well plate to the assay plate. Plates were sealed and incubated for 18 to 24 hours at 2°C to 8°C, then washed as described above. Biotinylated goat anti-guinea pig IgG antibody (Vector Labs, #BA-7000) was diluted in assay buffer, added to assay plates, and plates were incubated for one hour at RT. ExtrAvidin®-Peroxidase (Sigma-Aldrich, #E2886) was diluted in assay buffer, added at 100 µL per well, and plates were incubated for one hour at RT. 100 µL per well of detection reagent 3,3ʹ,5,5ʹ-tetramethylbenzidine (Sigma-Aldrich, #T0440) was allowed to develop for 15 minutes in the dark at RT. Absorbance was measured at 450 nm with a SpectraMax® M2 microplate reader (Molecular Devices, LLC).

Statistical analysis on immunological results were completed using GraphPad Prism 6 software. A one-way anova was used to identifiy significant differences between study groups. Tukey’s multiple comparisons test was used to compare group means if a significant difference was observed using an alpha value of 0.05. A *p*-value of less than 0.001 was considered significant.

## Results

### Formulation development

**Figure 1.**
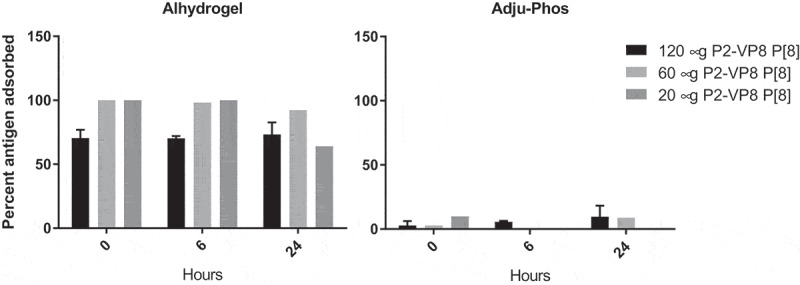
Percent adsorption of P2-VP8* P[8] protein to aluminum adjuvants in 0.9% (w/v) saline. Adsorption was measured using the bicinchoninic acid assay (n = 3 at each time point).

Monovalent P2-VP8* P[8] antigen adsorption to aluminum adjuvants Adju-Phos and Alhydrogel is shown in Figure 1. Approximately 70% of monovalent antigen adsorbed at all concentrations tested to Alhydrogel within 30 minutes, including near complete adsorption at antigen concentrations of 20 and 60 µg/mL, with a constant concentration of aluminum of 1.125 mg/mL. In contrast, little to no antigen adsorption was observed to Adju-Phos over the same time period at the antigen concentrations tested. Adsorption to Adju-Phos increased modestly (~10% additional antigen) after further incubation overnight. Due to the high antigen adsorption observed with Alhydrogel, this adjuvant was selected for further formulation experiments and a concentration of 1.125 mg Al/mL was selected.

**Figure 2.**
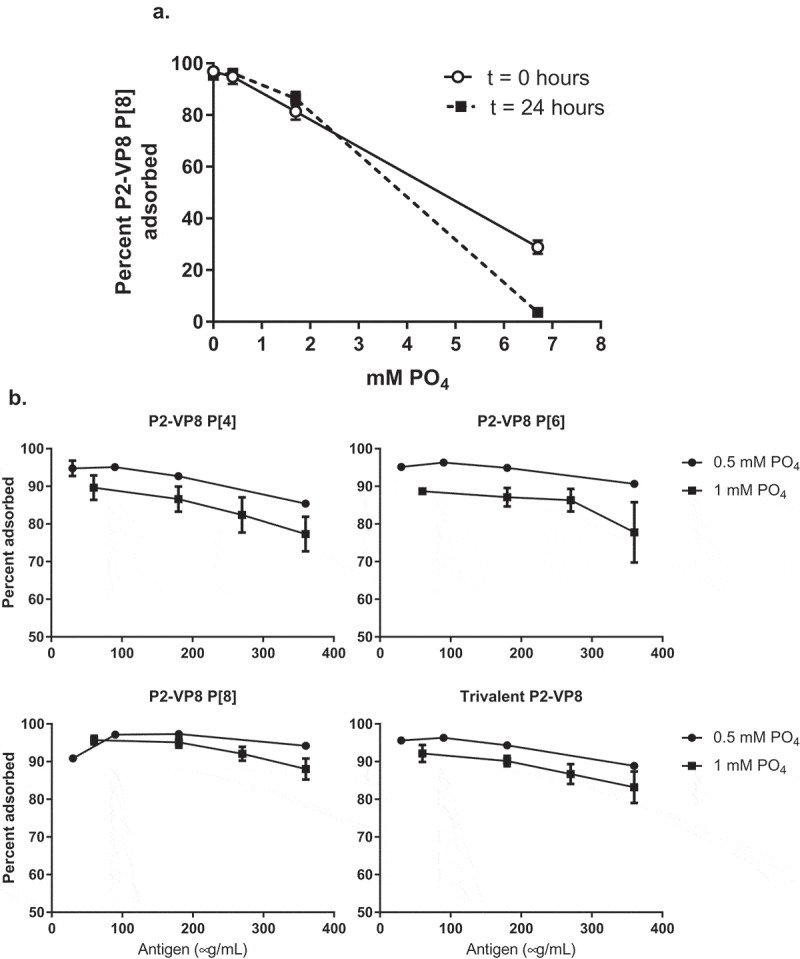
Influence of phosphate (PO_4_) concentration in buffer on P2-VP8* monovalent and trivalent adsorption to Alhydrogel, using the bicinchoninic acid assay. (a) The percent adsorption of monovalent P2-VP8* P[8] (120 µg/mL) to Alhydrogel (1.125 mg Al/mL) at different concentrations of PO_4_ buffer with 0.9% saline, pH ~7.2, was determined at time of formulation (circles) and after 24-hour incubation at 2°C to 8°C (squares) (n = 3 measurements). (b) The percent adsorption of P2-VP8* monovalent and trivalent antigens in 0.5 mM and 1 mM PO_4_ buffer for antigen concentrations ranging from 30 to 360 µg/mL. Values are the means ± standard deviations (n = 3 measurements).

The adsorption of P2-VP8* P[8] to Alhydrogel over a range of PO_4_ concentrations was measured at two time points (t = 0 and 24 hours) using the BCA assay (Figure 2a). Monovalent and trivalent antigen adsorption was then determined using both 0.5 mM and 1 mM PO_4_-containing buffers (Figure 2b), with results showing that a buffer strength of less than 1 mM PO_4_ was required to maintain greater than 90% antigen adsorption to Alhydrogel, especially as protein concentrations increased. A buffer concentration of 0.5 mM PO_4_ was selected for all subsequent formulation and stability studies to ensure a high adsorption to Alhydrogel.

#### Trivalent formulation characteristics and stability

Table 1 summarizes the target attributes for the final trivalent formulation. The trivalent P2-VP8* formulation met all target requirements for a stability study of 48 weeks at 2°C to 8°C, demonstrating a product shelf life of approximately one year at this temperature.

**Table 1.** Trivalent P2-VP8* target formulation attributes.

Assay	Target attribute
Appearance	White suspension; clear supernatant after centrifugation
Antigen identity^a^	3 separate peaks on chromatogram that align with monovalent and trivalent standards
pH	6.90 ± 0.30
Percent antigen adsorption to Alhydrogel	>90% antigen adsorbed (supernatant/total) × 100%
Antigen integrity^b^	2 bands near 20 kDa (upper band: P[6], lower band: P[4] and P[8])
Total protein content^c^	360 ± 72 µg/mL
Aluminum content (Alhydrogel)^d^	1.125 ± 0.02 mg Al/mL

^a^Antigen identity in the trivalent formulation was confirmed by ion exchange high-performance liquid chromatography.

^b^Antigen integrity was assessed by sodium dodecyl sulfate polyacrylamide gel electrophoresis of desorbed antigen.

^c^Total protein was determined by A280: absorbance at 280 nm for t = 0 and bicinchoninic acid assay for t = 48 weeks.

^d^Aluminum content was determined by ethylene diamine tetra-acetic acid chelation assay.

**Figure 3.**
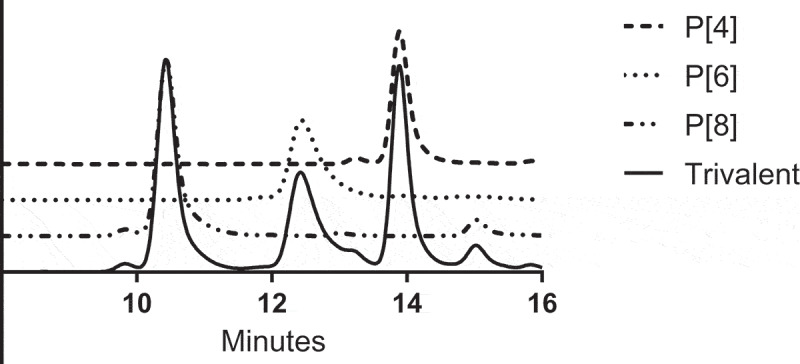
Ion exchange high-performance liquid chromatography data comparing monovalent standard elution peaks P[4], P[6], and P[8] with that of the trivalent vaccine antigen.

Antigen identity in the trivalent formulation was confirmed by IEX-HPLC, as shown in Figure 3. Analysis of the IEX-HPLC chromatograms for the desorbed sample compared with the monovalent standards confirm the identity of all three antigens in the trivalent vaccine sample. The elution times for the representative monovalent P2-VP8* peaks consistently matched the expected values and no anomalous peaks were noted after comparison of the sample chromatogram with the trivalent control.

**Figure 4.**
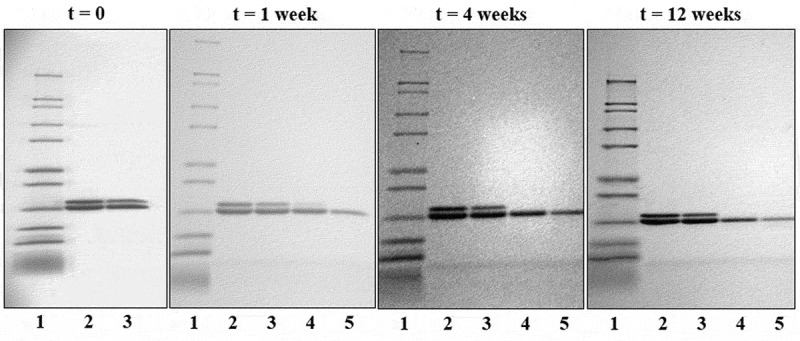
SDS-PAGE images of the trivalent formulation at t = 0 and after storage at different temperatures for one, four, and 12 weeks. The lanes are as follows: (1) ladder; (2) trivalent standard; (3) whole vaccine at 2°C to 8°C; (4) whole vaccine at 25°C; and (5) whole vaccine at 37°C. Double bands representing the three antigens (upper: P[6] and lower: P[4] and P[8]) are at approximately 20 kDa. Reduction in band intensity over time is due to incomplete desorption of antigen from adjuvant particles related to increased strength of antigen binding to adjuvant.

Antigen integrity was assessed by SDS-PAGE after desorbing the trivalent proteins from the adjuvant. Figure 4 (t = 0) shows two bands at 20 kDa, corresponding to the three vaccine antigens. P[8] and P[4] are almost identical in size and appear as the overlapping lower band and P[6] appears as the slightly higher, top band.

### Stability studies

Antigen integrity was monitored by SDS-PAGE for samples stored at 2°C to 8°C, 25°C, and 37°C for up to 12 weeks (Figure 4). Two bands at approximately 20 kDa represent the trivalent formulation. After one week of storage at elevated temperatures, a fading of the top band, representing the P[6] antigen, was observed by SDS-PAGE. This band was not visible at the four- or 12-week time point for samples stored at 25°C or 37°C, indicating either incomplete desorption of the antigen from the adjuvant, protein loss, or protein degradation.

**Figure 5.**
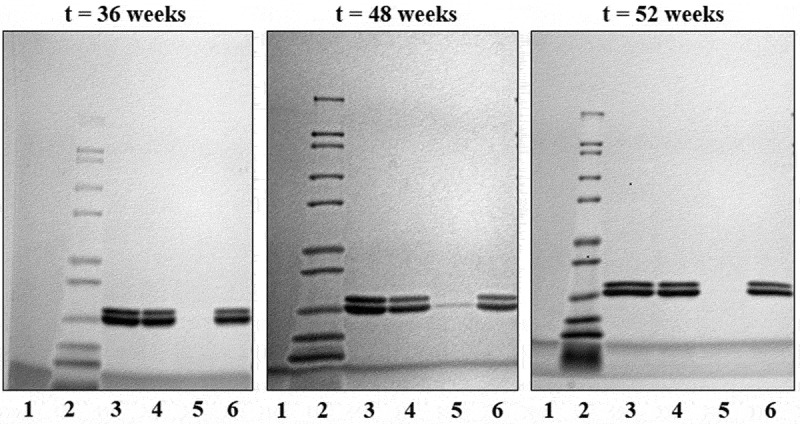
SDS-PAGE images of the trivalent formulation during storage at 2°C to 8°C. The lanes are as follows: (1) blank; (2) ladder; (3) trivalent standard; (4) whole vaccine; (5) vaccine supernatant; and (6) vaccine pellet. Double bands representing the three antigens are at approximately 20 kDa.

Long-term stability samples were stored at 2°C to 8°C for up to one year. Test samples were centrifuged to collect the supernatant and pellet for analysis at 36, 48, and 52 weeks. SDS-PAGE analysis of the desorbed trivalent formulation, shown in Figure 4, results in two visible bands, the lower of which represent both the P[4] and P[8] antigens (20.4 kDa and 20.3 kDa) while the higher molecular weight band represents the P[6] antigen (20.6 kDa). Long-term storage at elevataed temperatures resulted in reduced desorption of the antigens from the Alhydrogel particles, resulting in weaker bands, particularly for the higher band (P[6]). Based on this observation, long-term stability samples were subjected to a modified desorption method by treatment with SDS and PO_4_ prior to SDS-PAGE analysis. Analysis of the long-term stability samples demonstrated that the antigens remained bound to the adjuvant (pellet) after storage at 2°C to 8°C for one year (Figure 5, lane 6) and were not degraded. A faint band was observed at the t = 48-week time point, but this was attributed to well spillover, which was confirmed by a follow-up measurement at 52 weeks showing that the vaccine integrity was maintained (Figure 5).

This finding led to repeat analysis of the elevated temperature samples using the BCA method to determine if the antigens were lost, degraded, or remained tightly bound to the adjuvant, which resulted in the apparent loss of antigen. Results of the BCA testing are shown in Table 2 and demonstrated that no loss of protein occurred during storage at elevated temperatures.

**Table 2.** Trivalent P2-VP8* antigen adsorption to Alhydrogel.

Time stored (weeks)	Total protein content^a^ µg/mL (percent bound to Alhydrogel)		
	28°C to 8°C	25°C	37°C
4	399 (94)	387 (99)	393 (98)
12	436 (96)	418 (98)	394 (99)
48	386 (96)	Not tested	Not tested

^a^ Protein content was determined by bicinchoninic assay.

Trivalent P2-VP8* protein content and antigen adsorption to Alhydrogel was measured throughout the study using the BCA method. Protein content of samples was stable at all time points and temperatures tested (Table 2). Samples of whole vaccine, pellet, and supernatant were tested at each time point. Antigen was not detectable in the supernatant. Due to the high percentage of antigen adsorption to Alhydrogel, the whole vaccine and pellet measurements were identical; results for the pellet are shown in Table 2. Antigen adsorption to Alhydrogel also remained stable for all times and temperatures tested, based on the amount of antigen detected in the pellet at all time points. The target attribute for antigen bound to aluminum of >90% (Table 1) was achieved.

## Immunogenicity in guinea pigs

### Adjuvant effect

**Figure 6.**
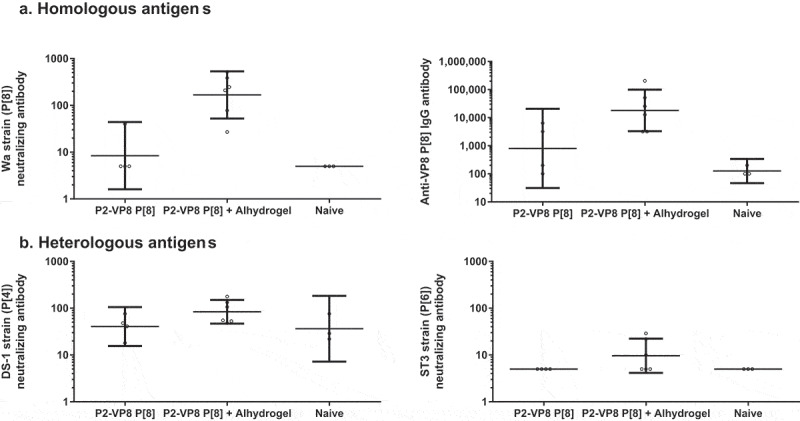
Serum antibody responses after intramuscular injections with P2-VP8* P[8] antigen with and without Alhydrogel adjuvant. Six animals for each antigen group with and without adjuvant were immunized on days 0, 14, and 28, and blood samples were analyzed on day 42 (two weeks after the final immunization). Three naïve animals for each antigen group were included as negative controls, and blood samples were analyzed on day 42. (a) Neutralizing antibodies and total binding immunoglobulin G (IgG) detected against homologous antigen. (b) Neutralizing antibody responses generated against heterologous antigens. Geometric mean titers with 95% confidence intervals are shown.

The immunogenicity of monovalent P2-VP8* P[8] antigen with and without Alhydrogel adjuvant was assessed in guinea pigs. Neutralizing and total binding IgG antibody responses are shown in Figure 6. The adjuvant provided an increase in neutralization titer against the homologous P[8] antigen, Wa strain. Neutralizing antibody responses to heterologous P[4] (DS 1 strain) and P[6] (ST3 strain) were modestly increased in the groups immunized with the adjuvanted vaccine.

Two animals died during the course of the in vitro studies. One animal that received antigen without adjuvant died on day 41, and one animal that received antigen with adjuvant died on day 42; thus, these subjects were not available for the post-immunization assessment. The necropsies showed intestinal intussusception, lung atelectasis, and bloated cecum filled with feces. Gastrointestinal disease is a common problem with guinea pigs and the veterinarian on staff determined both deaths were unlikely to be related to the study methods.^30^

### Effect of tetanus toxoid P2 epitope on immunogenicity

**Figure 7.**
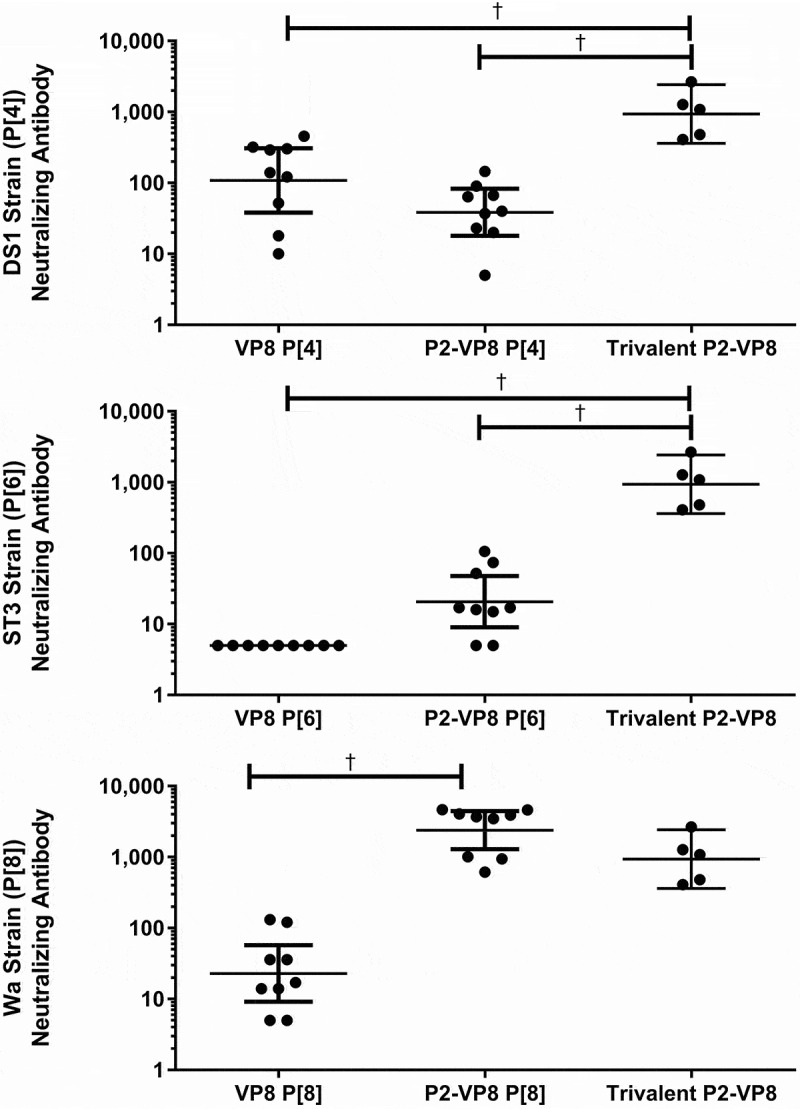
Serum neutralizing antibody responses (y-axis) after intramuscular injections with P2-fused and non-fused monovalent antigens with Alhydrogel adjuvant (x-axis). Animals were immunized on days 0, 14, and 28, and blood samples were analyzed on day 42 (two weeks after final immunization). Neutralizing antibody results against DS-1 (P[4]), ST3 (P[6]), and Wa (P[8]) rotavirus strains. Geometric mean titers with 95% confidence intervals are shown.

^†^Statistically significant *p*-values< 0.001 between group means using Tukey’s multiple comparisons test.

**Figure 8.**
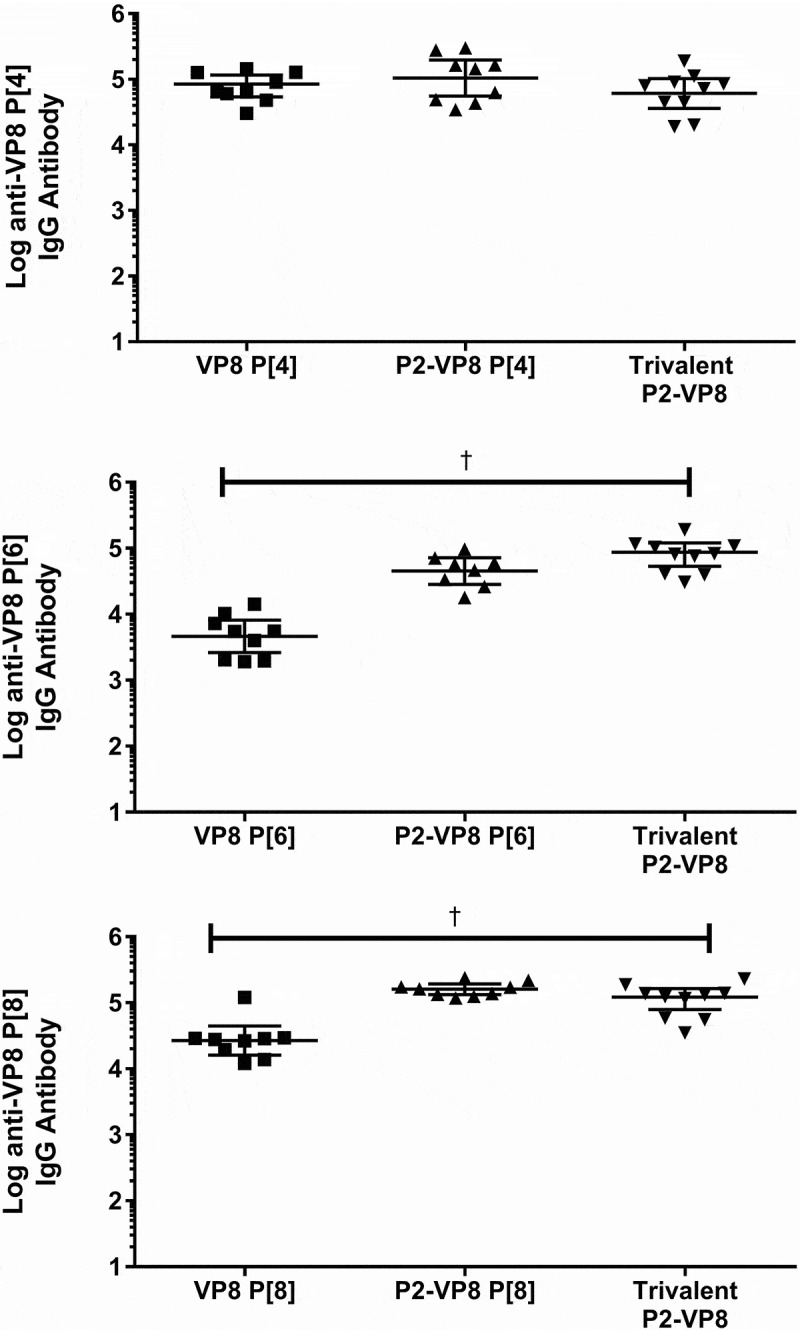
Serum immunoglobulin G (IgG) antibody responses after intramuscular injections with P2-fused and non-fused monovalent antigens adsorbed to Alhydrogel adjuvant (x-axis). Animals were immunized on days 0, 14, and 28, and blood samples were analyzed on day 42 (two weeks after final immunization). Binding immunoglobulin G results against VP8* and P2-VP8* antigens are shown as geometric mean titers with 95% confidence intervals.

^†^Statistically significant *p*-values< 0.001 between group means using Tukey’s multiple comparisons test.

The effect of fusing VP8* monovalent antigens with the P2 epitope for tetanus toxoid on immunogenicity was evaluated using the guinea pig model. Animals were immunized with adjuvanted monovalent antigens with or without fusion of the P2 epitope. Neutralizing antibody and total binding IgG responses to both homologous antigen and to the trivalent mixture are shown in Figures 7 and 8. Total binding IgG responses generated against both P2-fused and non-fused antigens were measured by two different ELISA methods detecting responses to either the P2-fused or non-fused antigens. Both ELISA methods were used to determine the effect the fusion had on the ability of the assays to measure an antibody response. No difference was observed between ELISA results from the P2-fused and the non-P2-fused antigens; only the P2-fused antigen results are shown in Figure 8. The P2-fusion had a modest positive effect on neutralizing antibody titers against the P[6] and P[8] antigens, while the fusion to the P[4] antigen showed little effect (Figure 7). The highest neutralizing antibody titer was generated in animals receiving the trivalent vaccine, rather than to any of the monovalent antigens alone, reaching statistical significace with a *p*-value <0.001 for all groups. For the total binding IgG results, immunization with the P2-fusion generated a statistically significant higher antibody response to the P[6] and P[8] antigens than immunization with the unfused antigens (Figure 8). The P2-fusion did not have any deleterious effects on immune responses generated to any of the VP8* antigens.

## Discussion

This manuscript reports the formulation work conducted in the development of a trivalent NRRV candidate composed of P2-VP8* recombinant proteins for P[4] DS-1, P[6] 1076, and P[8] Wa strains and adjuvanted with Alhydrogel, for parenteral administration. This work is an import step in the rotavirus vaccine field because it describes the formulation development of one of the most advanced NRRV candidates. While there are several live attenuated rotavirus vaccine on the market advancement of a NRRV candidate with delivery via the parenteral route is a significant step forward for rotavirus vaccine development.

Two potential adjuvants, Adju-Phos and Alhydrogel, were evaluated for use in this vaccine formulation. A higher percentage of antigen adsorption was observed with Alhydrogel (achieving > 95%) for the trivalent formulation compared to aluminum phosphate, where < 10% adsorption was observed even after 24 hours of incubation. This is likely due to the relative zeta potential of the Al adjuvant particles in relation to the zeta potential values of the trivalent formulation antigens. At the pH of adsorption (7.2), Alhydrogel would have a positive charge, and Adju-Phos a negative one.^31^ The three antigens have calculated isoelectric point values less than 7.2 and thus would have negative charges at the vaccine pH. The resulting effect would be an attraction to Alhydrogel adjuvant and a repulsion from the Adju-Phos adjuvant.

Previous studies have documented that higher concentrations of PO_4_ in a vaccine buffer may limit the amount of antigen adsorption to Alhydrogel.^32^ This is likely due to ligand exchange with the bound antigens or incorporation into the surface of the adjuvant particles, thus imparting a subsequent shift in the surface charge of the adjuvant (zeta potential), closer to that of Adju-Phos.^33^ Consistent with previous observations, a very low PO_4_ concentration of 0.5 mM was selected for the final vaccine formulation. The low PO_4_ concentration in the final vaccine formulation led to concerns with long-term stability; however, data from a separate ongoing study have demonstrated stability for at least two years at 2°C to 8°C (data not shown).

During the elevated temperature studies, loss of the P[6] antigen was observed after four weeks at both temperatures tested. Upon further evaluation, it was determined that this apparent loss was due to a lack of antigen desorption from the adjuvant and not to antigen loss or degradation. Repeat analysis of elevated temperature samples using the BCA method instead of the SDS-PAGE analysis showed consistent, measurable amounts of protein in the pellet, with little to no protein appearing in the supernatant, indicating the antigen remained bound to adjuvant at 2°C to 8°C for up to 48 weeks, and four weeks at 25°C and 37°C.

Preclinical immunogenicity studies in guinea pigs were conducted to show binding and neutralizing antibody responses to all rotavirus strains included in the vaccine. Vaccine immunogenicity against homologous and heterologous antigens was enhanced by the fusion of the recombinant protein constructs with the tetanus toxoid universal CD4 + T cell epitope P2. These formulation development and preclinical studies have been used to support further advancement of this vaccine candidate for human use. Animal studies were limited by the number of animals per study group (5 to 7 animals/group). Additional studies should be conducted to further confirm the conclusions made above. This work was limited not only by the animal study sizes mentioned above but also by the limited characterization of the animal model.

This vaccine continues to be evaluated in human trials, and has demonstrated safety and immunogenicity consistent with the guinea pig data presented here in a guinea pig toxicology study (unpublished data).^13,21,23,34,35^ In addition to the animal studies described here, additional studies have been published describing the potential mechanisms of parenteral rotavirus protection using animal models.^36,37^ Several studies have been conducted to characterize the vaccine candidate as well as on going work to correlate in vitro analysis methods and in vivo guinea pig immunogenicity (manuscripts in preparation: McAdams D and White JA).^38,39^ In addition to the clinical advancement of this vaccine candidate, the formulation developed is undergoing a long-term controlled stability study to support drug labeling and regulatory filing in the future.

## Abbreviations

BCA: bicinchoninic acid
BSA: bovine serum albumin
DPBS: Dulbecco’s phosphate buffered saline
E. coli: Escherichia coli
EDTA: ethylenediaminetetraacetic acid
ELISA: enzyme-linked immunosorbent assay
IEX-HPLC: ion exchange high-performance liquid chromatography
IgG: immunoglobulin G
NRRV: nonreplicating rotavirus vaccine
PO_4_: phosphate
RT: room temperature
SDS-PAGE: sodium dodecyl sulfate polyacrylamide gel electrophoresis
